# Memory consolidation effects on memory stabilization and item integration in older adults

**DOI:** 10.3758/s13423-016-1197-0

**Published:** 2016-11-23

**Authors:** Helen Brown, Elizabeth A. Maylor

**Affiliations:** Department of Psychology, University of Warwick, Coventry, CV4 7AL UK

**Keywords:** Aging, Memory consolidation, Memory stabilization, Item integration, Vocabulary learning

## Abstract

This study examined the differential effects of aging on consolidation processes that strengthen newly acquired memory traces in veridical form (*memory stabilization*) versus consolidation processes that are responsible for integrating these memory traces into an existing body of knowledge (*item integration*). Older adults learned 13 nonwords and were tested on their memory for the nonwords, and on whether these nonwords impacted upon processing of similar-sounding English words immediately and 24 hours later. Participants accurately recognized the nonwords immediately, but showed significant decreases in delayed recognition and recall. In comparison, the nonwords impacted upon processing of similar-sounding words only in the delayed test. Together, these findings suggest that memory consolidation processes may be more evident in item integration than memory stabilization processes for new declarative memories in older adults.

**Electronic supplementary material:**

The online version of this article (doi:10.3758/s13423-016-1197-0) contains supplementary material, which is available to authorized users.

When a new piece of information is encountered, it must not only be encoded into short-term memory but must also establish a representation in long-term memory if it is to be retrieved later. This process is commonly referred to as memory consolidation. There is evidence that memory consolidation processes occur during both periods of quiet wakefulness (e.g., Dewar, Alber, Butler, Cowan, & Della Sala, 2012; Werchan & Gomez, 2013) and periods of sleep (see Rasch & Born, 2013, for a review). While memory consolidation processes during periods of wake and sleep may differ, the focus of the current study is on the differential effects of aging on the stabilization of new memory traces versus the integration of these memory traces into long-term memory over a 24-hour consolidation period.

Memory consolidation research in older adults has largely focused on processes that occur during periods of sleep, given that there are clear age-related changes in sleep quality and quantity (Hornung, Danker-Hopfe, & Heuser, 2005). However, despite the growing interest in the effects of aging on memory consolidation processes in recent years (see Scullin & Bliwise, 2015, for a review), there remain some outstanding questions.

The effects of aging on the consolidation of procedural (skill-based) memory appear to be relatively clear, with older adults typically showing an absence or reduction of postsleep improvements in tasks measuring procedural memory compared to young adults (Scullin & Bliwise, 2015). In comparison, the effects of aging on the consolidation of declarative (fact/event) memory are less clear. Some studies examining the consolidation of declarative memory during sleep find similar effects in young and older adults (Aly & Moscovitch, 2010; Wilson, Baran, Pace-Schott, Ivry, & Spencer, 2012). Other studies indicate an absence/reduction of declarative memory consolidation effects in older but not young adults (Cherdieu, Reynaud, Uhlrich, Versace, & Mazza, 2014; Mander et al., 2013; Scullin, 2013). Also, one recent study reports evidence of consolidation only in high performing older adults (Sonni & Spencer, 2015).

Moreover, few studies have examined memory consolidation effects on integration processes (the incorporation of newly learned information into preexisting schema) in older adults. Stickgold (2013) distinguished integration processes from consolidation processes that merely serve to strengthen and stabilize memories in veridical form, which have already been studied in older adults (with mixed results; see above) using tasks such as word or sentence recall (e.g., Giambra & Arenberg, 1993), word-pair learning (e.g., Scullin, 2013), or object location learning tasks (e.g., Sonni & Spencer, 2015). Integration is critically different from memory stabilization in that it requires newly learned pieces of information to be integrated into an existing body of knowledge.

As far as we are aware, only one study has examined memory consolidation effects on memory integration in older adults, and this study examined multi-item generalization (e.g., gist extraction, rule extraction, false memories) rather than item integration (e.g., vocabulary learning): Lo, Sim, and Chee (2014) induced false memories by teaching participants lists of semantically related words. At test, participants indicated whether words had previously been studied in an old/new recognition task. Lo et al. found that sleep (as compared to an equivalent period of time awake) significantly reduced false recognition of semantically related lures. This finding is consistent with similar studies examining false memories using recognition tests in young adults (e.g., Fenn, Gallo, Margoliash, Roediger, & Nusbaum, 2009), which have also shown reduced false memory levels after sleep. Together, these findings suggest that sleep-associated memory consolidation processes contribute to the reduction of false memory formation in a similar way across young and older adults. However, the false memory literature in young adults is complicated somewhat by findings from studies that use recall (rather than recognition) tests. Contrary to the findings reported above, studies using recall tasks at test have shown that false memories are preferentially preserved by a consolidation period that includes sleep (e.g., Payne et al., 2009). Thus, it is vital to use identical test procedures wherever possible when comparing different age groups. Crucially, memory consolidation effects on item integration have yet to be tested in older adults.

In this study, we examined the effect of memory consolidation processes on both memory stabilization and item integration in older adults. We used vocabulary learning paradigms that have already shown robust memory consolidation effects (memory stabilization *and* item integration) in young adults (e.g., Dumay & Gaskell, 2007) and children (e.g., Henderson, Weighall, Brown, & Gaskell, 2012). In these studies, participants were taught new words that differed from existing English words only in the final vowel and consonant (e.g., *dolph* *eg* *–dolph* *in*). After learning the nonwords, participants were tested (immediately and after a delay) on whether they could recall and recognize the studied nonwords, and also on whether learning the nonwords affected processing of the similar-sounding existing English words. Slower processing of existing words with new competitors (as compared to control words without new competitors) indicates that the studied nonwords have been integrated with existing knowledge and are engaging in lexical competition processes with similar sounding words during spoken word recognition (cf. Marslen-Wilson & Zwitserlood, 1989).

Previous studies using this paradigm in young adults and children (e.g., Dumay & Gaskell, 2007; Henderson et al., 2012) have revealed accurate recognition of studied nonwords immediately after study. Recall of the studied nonwords was poorer immediately, but improved significantly after a delay, provided that the consolidation period included sleep. Moreover, evidence that the studied nonwords affected processing of similar sounding existing words only emerged at delayed test points that followed a consolidation period that contained sleep. Thus, in young adults and children, memory consolidation processes, especially those associated with sleep, appeared to both stabilize and strengthen the representations of the nonwords, and also contributed to the integration of these representations into a long-term store of vocabulary knowledge where similar-sounding words affect processing of each other.

This pattern of results has been replicated several times in both young adults (e.g., Dumay & Gaskell, 2007; Gaskell & Dumay, 2003) and children (e.g., Brown, Weighall, Henderson, & Gaskell, 2012; Henderson, Weighall, Brown, & Gaskell, 2012, 2013) using different tasks to measure lexical integration (lexical decision, pause detection) and memory for the nonwords themselves (cued recall, free recall, two-alternative forced choice recognition, old/new recognition). The same pattern of results has also been found using different sets of stimuli—for example, words derived from existing English words by changing the final vowel and consonant, as in *dolphin/dolpheg* (e.g., Gaskell & Dumay, 2003) versus words derived from existing English words by adding a consonant cluster to the end of the word, as in *shadow/shadowks* (e.g., Dumay & Gaskell, 2007). Thus, we already know that for young adults and children the effects are reliable and replicable. As such, we have not collected additional data from young adults and children in this study. Instead, our approach was to test a group of healthy older adults using identical stimuli,^1^ tasks, procedures, and data analysis techniques to Henderson et al. (2013), who examined vocabulary learning in both young adults and children, allowing us to draw direct comparisons with the data reported in that study.

With regard to memory stabilization effects (measured via a cued recall task and a two-alternative forced choice recognition task), the predictions are unclear. Given that previous research provides conflicting results, we might predict either that older adults will show the same pattern as young adults and children (cf. Aly & Moscovitch, 2010; Wilson et al., 2012), or that older adults, unlike young adults and children, will not show evidence of memory stabilization at the delayed test point (cf. Cherdieu et al., 2014; Mander et al., 2013; Scullin, 2013). As for item integration, no study to date has examined the effects of memory consolidation on this process in older adults. However, results from studies examining multi-item generalization suggest that memory consolidation processes may have similar effects on this type of memory integration in young and older adults. Based on this finding, we predict that older adults (like young adults and children) will show evidence of item integration only in the delayed test session.

## Method

### Participants

Thirty-six older adults (13 male), aged 61–78 years (*M* = 70.3, *SD* = 4.4), were recruited from the Warwick Age Study Panel (all living independently in the community) and were rewarded with payment. Participants were monolingual native English speakers with normal or corrected-to-normal vision and hearing. None of the participants had previously had a stroke or head injury, and none reported having a language, learning, or sleep disorder. Participants were instructed not to take daytime naps for the duration of the study. Informed consent was obtained prior to the first session.

To assess cognitive functioning, participants completed the Digit Symbol Substitution Test (DSST) from the *Wechsler Adult Intelligence Scale–Revised* (Wechsler, 1981) as a measure of processing speed, and the multiple choice part of the Mill Hill vocabulary test (Raven, Raven, & Court, 1988) to provide a measure of crystallized intelligence.^2^ The mean scores were 50.0 (*SD* = 13.3) in the DSST and 24.2 (*SD* = 4.7) in the Mill Hill vocabulary test. Both of these scores are typical for samples of healthy older adults with a mean age of 70 years (cf. Badham, Hay, Foxon, Kaur, & Maylor, 2016; Badham & Maylor, 2016). For example, Hoyer, Stawski, Wasylyshyn, and Verhaeghen’s (2004) meta-analysis of DSST data from 3,876 healthy adults aged 61–79 years revealed a comparable overall mean of 48.6.

### Stimuli

The main stimuli consisted of 26 word triplets, each containing one existing word and two nonwords, taken from Henderson et al. (2013, Experiment 2). Existing words were monomorphemic with uniqueness points located at or before the final vowel. Nonwords differed from the existing word at the final vowel (e.g., *dolpheg* */dolph* *i* *n*) and from each other at the final consonant or consonant cluster (e.g., *dolphe* *g* */dolphe* *ss*). One nonword was learned in the phonics-based tasks. The other served as a distracter in the two-alternative forced choice (2AFC) task. All three words had the same stress pattern. The stimulus triplets were divided into two lists of 13, with existing words matched for age of acquisition, number of syllables and phonemes, and frequency (see Brown et al., 2012, for more detail). Participants learned one list of 13 nonwords during the phonics-based tasks, with list counterbalanced across participants. The remaining list served as a control set of items in the test of lexical competition.

### Design and procedure

Participants completed two experimental sessions on consecutive days, with the two sessions occurring approximately 24 hours apart (*M* = 23.8; range: 20.3–25.0). The tasks completed in each session are summarized in Table 1 and are described in more detail below. In the first session, participants were exposed to 13 nonwords in phoneme monitoring and phoneme segmentation tasks. Participants then completed a measure of lexical competition (pause detection) followed by two measures of memory for the nonwords themselves (a cued recall task and a 2AFC task). In the second session, participants completed only the pause detection, cued recall, and 2AFC tasks, with the tasks fixed in this order. At the end of Session 2 participants also completed a morningness-eveningness questionnaire (MEQ; Horne & Östberg, 1976), which provides a subjective measure of the time of day at which a person is most alert.^3^ Participants were tested individually in either a University laboratory (*N* = 16), or in a quiet room in their home (*N* = 20).^4^ For all participants, both sessions took place in the same location. Experimental tasks were administered using a Toshiba laptop, with stimuli presented binaurally over headphones at a comfortable listening level.

**Table 1:** Tasks completed in Sessions 1 and 2 (S1 and S2) with descriptions of each task using the nonword *dolpheg* as an example

Task	S1	S2	Task description
Phoneme monitoring	✓		Does *dolpheg* contain a prespecified sound? (e.g., /d/–yes, /s/–no)
Phoneme segmentation	✓		What is the first/last sound in *dolpheg?*
Pause detection	✓	✓	Does the word *dolphin* contain a short pause?
Cued recall	✓	✓	Which nonword started with *dol-?*
Two-alternative forced choice	✓	✓	Which of these nonwords did you learn during the earlier tasks: *dolpheg* or *dolphess*
Morningness–eveningness questionnaire		✓	Standardized questionnaire

In the *phoneme monitoring task,* participants were exposed to one list of 13 nonwords, indicating via a button-press response whether a specified phoneme was present/absent on each trial. The task began with five real-word practice trials. Experimental trials were split into six blocks, with the target phonemes /p/, /t/, /d/, /s/, /m/, and /b/ presented in a fixed order. Each nonword occurred twice per block, resulting in 12 exposures to each nonword. The order of the nonwords was randomized in groups of 13 (i.e., one full repetition of the list). Instructions emphasized accuracy.

In the *phoneme segmentation task,* participants listened to the same 13 nonwords and were asked first to repeat them aloud, and then to say just the first sound (Block 1) or last sound (Block 2) in the word. Items were repeated three times per block, resulting in an additional six exposures to each nonword during this task. Accuracy was recorded.

Following training, participants completed the *test of lexical competition* (pause detection; Mattys & Clark, 2002; Mattys, Pleydell-Pearce, Melhorn, & Whitecross, 2005): Participants monitored for 200-ms pauses that were artificially inserted into words. This task has been shown to be a sensitive measure of lexical processing in both children (Henderson et al., 2013) and young adults (e.g., Gaskell & Dumay, 2003; Mattys & Clark, 2002) although it has not, to our knowledge, been used with older adults before. During the pause detection task, participants heard all 26 existing words (13 test words with a novel competitor from the trained list, 13 control words without a new competitor from the control list), and 26 filler words (all bi- or trisyllabic monomorphemic English nouns, taken from Henderson et al., 2013). Half of the test words, half of the control words, and half of the filler words contained pauses. Four experimental lists were created to allow each experimental word to be equally represented in all four cells of the design (Test/Control × Pause Present/Pause Absent). The pause detection task began with five practice trials, followed by all 26 experimental words and 26 filler words presented in a randomized order. Participants indicated via a button-press response whether a pause was present in each word. For the 26 experimental words, pauses were inserted just before the final vowel offset if the following consonant was a voiceless plosive, and just after the second vowel offset otherwise (following Dumay & Gaskell, 2007; Henderson et al., 2012, 2013). Pauses were inserted at various points in the filler words. Response times (RTs) were measured from word onset. The intertrial interval was 1 s. Instructions emphasized both speed and accuracy.

Finally, we tested participants’ memory of the studied nonwords. Participants first completed a *cued recall task* in which they heard the first CVC syllable of the studied nonwords (e.g., *dol…*), presented in a randomized order, and tried to complete the cue with one of the studied nonwords. Cues could be replayed as many times as required. If participants produced existing words (e.g., *dolphin*) they were reminded that their task was to try to recall the nonwords from the training tasks and were given another chance to respond. Second, participants completed a 2AFC task in which they heard both a studied nonword (e.g., *dolpheg*) and its corresponding unstudied foil (e.g., *dolphess*). Participants were instructed to listen to both items and then to indicate via a button-press response which nonword had been heard previously in the phonics-based tasks. All 13 nonwords from the training list, and their corresponding foils, were presented in a randomized order, with the order of the two items in each trial pseudorandomized across participants. Instructions in both of these tasks emphasized accuracy.

## Results

All data were analyzed in accordance with the methods used by Henderson et al. (2013), allowing us to draw direct comparisons between the performance of older adults, and that of young adults and children in the same tasks (see Table 2). For the pause detection task, the analysis compared RTs to test and control words in each session. For the cued recall and 2AFC recognition tasks, accuracy between sessions was compared. For all test tasks, list was included in the analyses as a between-participants factor, but as it was not involved in any main effects or interactions, it will not be reported here. (The statistics for the factor *list,* and interactions involving this factor, are reported in full in the Supplementary Materials.) In addition to standard null hypothesis tests, an estimated Bayes factor (*BF*_*10*_, obtained from JASP computer software; Love et al., 2015), based on model averaging, was calculated. *BF*_*10*_ provides an odds ratio for the alternative/null hypothesis. Values <1 favor the null hypothesis while values >1 favor the alternative hypothesis.

**Table 2:** Mean percentage correct (and *SDs*) in each session for the cued recall and two-alternative forced choice (2AFC) tasks, and mean RTs (ms, and *SD*s) in each session for test (items with novel nonword competitors) and control (items without novel nonword competitors) words in the pause detection task

			Henderson et al. (2013)			
	Older adults		Young adults^1^		Children^2^	
Measure	Session 1	Session 2	Session 1	Session 2	Session 1	Session 2
Cued recall (%)	21.2 (17.4)	14.3 (14.5)	54.3 (23.4)	74.8 (18.2)	17.5 (17.2)	60.7 (20.4)
2AFC (%)	91.0 (9.8)	86.5 (14.6)	91.3 (10.6)	98.7 (3.9)	68.8 (23.2)	85.9 (16.1)
Pause detection RT (ms) ^3^						
Test word	1198 (219)	1168 (197)	726 (166)	773 (206)	1300 (254)	1353 (252)
Control word	1180 (214)	1137 (188)	772 (209)	738 (217)	1296 (291)	1247 (269)
Difference	*ns*	*p* < .05	*p* = .07^4^	*p* = .08	*ns*	*p* < .05

*Note.* Comparable data for young adults and children, taken from Henderson, Weighall, Brown, and Gaskell (2013), are also reported for comparative purposes.

^1^ *N* = 18; *M*_age_ = 19.3 years (*SD* = 0.7).

^2^ *N* = 18; *M*_age_ = 7.9 years (*SD* = 0.3).

^3^There was a significant interaction between session and word type for both age groups reported in Henderson et al.: young adults, *F*(1, 16) = 9.61, *p* = .007, η_p_^2^ = .38; children, *F*(1, 16) = 5.70, *p* = .03, η_p_^2^ = .26.

^4^In Session 1, young adults exhibited a marginally significant priming effect, with faster RTs for test compared to control words. This effect was most likely due to repeated exposure to the phonologically similar nonwords in the phonics-training tasks and subsequent priming of test words in the pause detection task.

### Training tasks

Mean percentage accuracy was 94.5 (*SD* = 3.0) in the phoneme monitoring task and 90.8 (*SD* = 28.9) in the phoneme segmentation task^5^ (first sound: *M =* 93.4, *SD* = 24.9; last sound: *M =* 88.2, *SD =* 32.3).

### Test tasks

In the *pause detection task,* only data from the 26 experimental words were included in the analysis. Data from incorrect responses (5.9 %) and extreme RTs (<200 ms [0 %] or >2.5 *SD*s from the condition mean [2.4 %]) were removed. RTs were averaged across pause-present and pause-absent trials (see Table 2 for means). A 2 (session) × 2 (word type: test vs. control) × 2 (list) repeated-measures ANOVA revealed a significant main effect of session, *F*(1, 34) = 7.09, *p* = .012, η_p_^2^ = .17, *BF*_*10*_ = 3.71, with RTs being shorter in the second session, most likely due to practice effects. The main effect of word type was marginally significant, *F*(1, 34) = 3.75, *p* = .061, η_p_^2^ = .01, *BF*_*10*_ = 0.66, indicating a trend toward faster responses to control relative to test words overall. Although the interaction between session and word type was not significant, *F* < 1, *BF*_*10*_ = 0.32, we conducted planned comparisons on the basis of Henderson et al. (2013; Table 2) showing significant slowing of test relative to control words in Session 2 but not in Session 1. Indeed, paired-samples *t* tests confirmed that while there was no significant difference between test and control words in Session 1, *t*(35) = 0.96, *p* = .344, *BF*_*10*_ = 0.45, the difference was significant in Session 2, *t*(35) = 2.22, *p* = .033, *BF*_*10*_ = 3.08,^6^ with participants responding slower to test than to control words at this later time point.

For both the *cued recall* and *2AFC* tasks*,* percentage correct in each session was entered into a 2 (session) × 2 (list) repeated measures ANOVA. This revealed a significant main effect of session in both tasks, *cued recall*: *F*(1, 34) = 11.84, *p* = .002, η_p_^2^ = .26, *BF*_*10*_ = 16.20; *2AFC*: *F*(1, 34) = 7.64, *p* = .009, η_p_^2^ = .18, *BF*_*10*_ = 4.24, indicating that there were significant decreases in both recall and recognition accuracy across sessions.

## Discussion

Older adults accurately recognized newly learned nonwords immediately after study, but showed significant decreases in both recognition and recall accuracy when tested 24 hours later. This contrasts with data from Henderson et al. (2013) showing that young adults and children significantly improved in recognition and recall at the 24-hour retest. The reduction in memory stabilization effects on memory for new words in older adults does not appear to be due to encoding difficulties. In Session 1, young and older adults had almost identical accuracy scores in the 2AFC task, and children and older adults had similar scores in the cued recall task. However, at the delayed test point, older adults showed significant decreases in recognition and recall while the young adults and children in Henderson et al. (2013) showed significant increases.

The absence of a memory stabilization effect for new declarative information in older adults, but not young adults and children, is consistent with a number of previous studies (e.g., Cherdieu et al., 2014; Mander et al., 2013; Scullin, 2013). Notably, the studies suggesting that there are similar consolidation effects on declarative memory in young and older adults (e.g., Aly & Moscovitch, 2010; Wilson et al., 2012) have found reduced performance in the delayed test relative to a baseline immediate test for both age groups. In these studies, evidence of memory stabilization came from the finding that participants performed better following a period of sleep compared to an equivalent period of time spent awake. Thus, memory stabilization effects in these studies were specific to sleep-associated memory consolidation processes. The current study did not differentiate between memory consolidation processes that occur during periods of wake versus sleep. Perhaps if we had compared two groups of participants, one tested after a delay that included sleep and another tested after remaining awake for an equivalent time period, we would have observed greater decreases in recognition and recall of the new words in the awake group. Such a finding would suggest that sleep plays a protective role in the stabilization of new vocabulary knowledge in older adults (although this would still be different from the enhancements seen in vocabulary knowledge in children and young adults following a delay that contains sleep). This is a question for future research.

One explanation as to why recall and recognition of nonwords decreased between sessions for older adults, but not young adults and children, may be that both the nonwords and their foils in the 2AFC task were repeated in both sessions. Dumay and Gaskell (2007) have previously demonstrated that there is no difference between pause detection and free recall performance for young adults who complete the 2AFC task at the end of each session and those who complete the 2AFC task only at the end of the final session, suggesting that reexposure effects are minimal for young adults. However, the inclusion of the 2AFC task in Session 1 may pose a larger problem for older adults as source memory is a function that is vulnerable to aging (e.g., Johnson, Hashtroudi, & Lindsay, 1993). That is, older adults may struggle to differentiate the target and foil nonwords in Session 2 after having heard both items in the 2AFC task in Session 1. Nonetheless, children also appear to have difficulties with source memory (e.g., Cycowicz, Friedman, Snodgrass, & Duff, 2001). Yet children (like young adults) show increases in recall and recognition of nonwords in the delayed test session. Thus, it seems unlikely that difficulties with source memory as a result of repeating the 2AFC test across sessions can fully account for the differences observed between the three age groups.

With regard to item integration, planned comparisons on the data from the pause detection task were indicative of lexical competition effects for older adults in the delayed, but not the immediate test. This was the pattern predicted on the basis of data observed in young adults and children (e.g., Henderson et al., 2013; see also Dumay & Gaskell, 2007; Henderson et al., 2012). Thus, memory consolidation processes appear to contribute to item integration in a similar manner across age groups, although the pattern is somewhat weaker in young and older adults compared to children (see Table 2). This is consistent with studies examining multi-item generalization, which have shown similar effects in young and older adults when the testing procedure is held constant across the two age groups (e.g., Fenn et al., 2009; Lo et al., 2014). Together, these findings suggest that memory consolidation processes contribute to memory integration in a similar way across the life span.

Taken together, the data suggest that there may be a dissociation between memory consolidation processes that contribute to memory stabilization and item integration in older adults. This suggestion is supported by a study by Tamminen, Payne, Stickgold, Wamsley, and Gaskell (2010) that found a dissociation between different aspects of consolidation during vocabulary learning in young adults (memory stabilization vs*.* item integration) and different aspects of sleep architecture—slow-wave sleep (SWS) and sleep spindles (11–15 Hz oscillations lasting up to 3 s). Specifically, greater SWS duration was associated with larger overnight decreases in RTs in an old/new recognition task (memory stabilization) while greater spindle activity was associated with larger overnight increases in lexical competition (item integration). However, research in older adults suggests that there is both a reduction in SWS quality and quantity (e.g., Ohayon, Carskadon, Guilleminault, & Vitiello, 2004; Scullin, 2013), and a reduction in the number of sleep spindles (e.g., Nicolas, Petit, Rompre, & Montplaisir, 2001) with increasing age. Given these findings, one might expect that item integration, like memory stabilization, should be reduced in older adults.

Alternatively, the observed dissociation between consolidation effects on memory stabilization and item integration processes in older adults could be related to the well-established finding that larger age differences are typically observed in tasks that require explicit retrieval, compared with tasks that depend on more automatic activation processes (e.g., Light, Prull, La Voie, & Healy, 2000, although the view that there are different effects of aging on explicit and implicit memory processes has recently been challenged by Ward, Berry, & Shanks, 2013). Thus, pause detection may depend on more implicit processes that are age invariant, in comparison with cued recall and recognition. To explore this possibility further, it would be necessary to measure both memory stabilization and item integration using tasks that tap implicit processes (e.g., perceptual identification—identification of words under noisy processing conditions—vs. pause detection).

To conclude, data from this study provide an important first step toward understanding the effects of aging on different aspects of memory consolidation (stabilization vs. item integration). Additional studies are required to differentiate between the consolidation processes that occur during sleep and wake states. For children and young adults, we already know that both the memory stabilization and item integration effects observed in Henderson et al. (2013) are specifically related to sleep-associated consolidation processes (e.g., Dumay & Gaskell, 2007; Henderson et al., 2012). Is the same true for older adults? And if so, is the dissociation between the consolidation effects on memory stabilization and item integration observed in the current study attributable to different aspects of sleep architecture, as suggested by Tamminen et al. (2010)?

## Electronic supplementary material

Below is the link to the electronic supplementary material.

ESM 1 (DOCX 27 kb)

## Footnotes

1 New audio recordings were produced by a female native speaker of English to account for differences in regional accents. Sound files were recorded using a Sony PCM-M10 audio recorder and were edited using audacity (http://audacityteam.org/).

2 Data from two participants were not available for these background measures.

3 Participants were tested at various times throughout the day. Twenty-three participants took part in the morning and 13 in the afternoon. There were no significant differences between participants who were tested in the morning and those who were tested in the afternoon in any of the training and test tasks (see Supplementary Materials for full details of these analyses). Moreover, scores from the MEQ suggested that most participants completed the study at optimal times of the day. Of the 23 participants tested in the morning, four had a definite morning preference, five had a moderate morning preference, 13 had neither a morning nor an evening preference, and one participant had a definite evening preference. Of the 13 participants tested in the afternoon, seven had a moderate morning preference and six had neither a morning nor an evening preference. In sum, only eight of the 36 participants were tested at a nonoptimal time of day, and in fact only one of these participants showed a strong preference for the opposite time of day. In summary, the time of day at which a participant was tested had little impact upon the pattern of results observed.

4 Location of testing was largely dictated by the availability of parking at the University during term time. Initial analyses of the data confirmed that location of testing did not influence the main pattern of results (see Supplementary Materials for full details of these analyses).

5 Occasionally, participants responded by saying the letter name rather than the first/last phoneme. When this happened, participants were reminded to focus on the sounds in the word rather than the spelling. However, letter names corresponding to the correct phoneme were scored as correct. In addition, some participants occasionally responded by saying more than one phoneme (typically CV for the first sound [e.g., “*do*” in “*dolpheg*”], and VC for the last sound [e.g., “*eg*” in “*dolpheg*”]). These responses were scored as correct if they accurately described the phonemes in the new words. However, participants were reminded that they should only say the very first or very last sound in the words.

6 According to Jarosz and Wiley (2014, Table 4), a *BF*_*10*_ factor between 3 and 10 indicates positive/substantial evidence for the alternative hypothesis.
