# Supplementary material for: Memory consolidation effects on memory stabilization and item integration in older adults
Source: Psychon Bull Rev. 2016 Nov 23;24(4):1032–9. doi: 10.3758/s13423-016-1197-0 (PMC5570810; doi:10.3758/s13423-016-1197-0)
Supplement: Supplementary file 1 — (DOCX 27 kb) [file 13423_2016_1197_MOESM1_ESM.docx]

**Supplementary Materials**

In addition to the main experimental variables, it is important to check that the results are not affected by other factors such as the list of items that the participant learned (List 1/List 2), the time of day at which participants were tested (morning/afternoon), or the location in which the test occurred (home/laboratory). Below we present analyses showing that these variables had little effect on the pattern of results reported in main paper.

**Stimulus List**

Each participant learned 13 nonwords from one of two lists. The lists were counterbalanced across participants, with an equal number of participants learning each list (*N* = 18). Given that the existing English words in each list were matched for Age of Acquisition, number of syllables, number of phonemes, and frequency (see Brown, Weighall, Henderson, & Gaskell, 2012, for more detail) we expected that performance in the training and test tasks would not differ as a function of which list of items the participant had learned.

***Training Tasks***

Mean accuracy in the phoneme monitoring task was 96.0% (*SD* = 2.6%) for List 1, and 93.0% (*SD* = 2.7%) for List 2. This difference was significant, *t*(34) = 3.44, *p* = .002, *BF_10_* = 18.79. Mean accuracy in the phoneme segmentation task was 93.1% (*SD* = 8.2%) for List 1, and 88.5% (*SD* = 8.6%) for List 2. This difference was not significant, *t*(34) = 1.65, *p* = .11, *BF_10_* = 0.93.

***Test Tasks***

There were no significant main effects or interactions involving list in any of the test tasks: *Pause detection* – list, *F* < 1, *BF_10_* = 0.35; list x session, *F* < 1, *BF_10_* = 0.26; list x word type, *F*(1, 34) = 3.03, *p* = .09, η_p_^2^ = .08, *BF_10_* = 0.48; list x session x word type, *F* < 1, *BF_10_* = 0.05. *Cued recall* – list, *F* < 1, *BF_10_* = 0.43; list x session, *F* < 1, *BF_10_* = 0.49. *2AFC* – list, *F*(1, 34) = 1.13, *p* = .30, η_p_^2^ = .03, *BF_10_* = 0.54; list x session, *F* < 1, *BF_10_* = 0.45.

***Summary***

There was no evidence that performance in the test tasks was affected by which list the participant had learned. This is despite the fact that participants were more accurate at performing the phoneme monitoring task for List 1 than for List 2.

Further examination of the data showed that there were two items in List 2 that produced accuracy scores < 20% for one of the target phonemes. First, accuracy was only 11.1% when participants were asked whether the phoneme */t/* occurred in the nonword *tulode* (which starts with the phoneme /tʃ/, not /t/). Second, accuracy was only 19.4% when participants were asked whether there was a /s/ in *parasheff* (which contains the phoneme /ʃ/, corresponding to the spelling *sh*)*.* In both cases, it is likely that participants were using their knowledge of how the phonologically similar English words were spelled to determine the answer. With trials corresponding to these portions of the dataset removed, the difference between lists was no longer significant, *t*(24) = 1.12, *p* = .27, *BF_10_* = 0.49. Importantly, errors that relate to phoneme-spelling confusion are unlikely to have had a large impact upon encoding of the nonwords, and as can be seen, had no subsequent impact upon performance in the test tasks.

**Time of Testing**

Participants completed the study at various time points across the day. Twenty-three participants completed sessions in the morning (*AM* group) and 13 completed sessions in the afternoon (*PM* group). Given evidence that the amount of time that elapses between learning and the onset of sleep may be critical for sleep-dependent consolidation (e.g., Gais, Lucas, & Born, 2006), it might be expected that performance would be better in Session 2 (or at least that there would be less forgetting) for participants who completed the training session in the afternoon.

***Training Tasks***

Mean accuracy in the phoneme monitoring task was 94.2% (*SD =* 3.3%) in the AM group, and 95.0% (*SD* = 2.5%) in the PM group. This difference was not significant, *t*(34) = -0.80, *p* = .43, *BF_10_* = 0.42. Mean accuracy in the phoneme segmentation task was 90.5% (*SD =* 8.8%) in the AM group, and 91.2% (*SD =* 8.5%) in the PM group. This difference was not significant, *t*(34) = -0.23, *p* = .82, *BF_10_* = 0.34.

***Test Tasks***

To examine whether time of test impacted upon performance in the test tasks, we re-ran the analyses reported in the main paper, but included time of test (AM/PM) as an additional between-participants variable in each analysis.

There was no overall effect of time of test in the *pause detection task*, *F* < 1, *BF_10_* = 0.23. However, the interaction between time of test and session approached significance, *F*(1, 32) = 3.10, *p* = .09, η_p_^2^ = .09, *BF_10_* = 0.41. Paired samples *t*-tests showed that RTs (in ms) decreased significantly between sessions for the PM group, *t*(12) = 3.75, *p* = .003, *BF_10_* = 16.75 (Session 1, *M* = 1226, *SD* = 157; Session 2, *M* = 1161, *SD* = 134), but not for the AM group, *t*(22) = 1.11, *p* = .28, *BF_10_* = 0.38 (Session 1, *M* = 1168, *SD* = 163; Session 2, *M* = 1148, *SD* = 123). Nonetheless, there was no interaction between time of test and word type, *F* < 1, *BF_10_* = 0.16, and time of test did not interact with the session by word type interaction, *F*(1, 32) = 1.24, *p* = .27, η_p_^2^ = .04, *BF_10_* = 0.05.

All main effects and interactions involving time of test were non-significant in the *cued recall* and *2AFC* tasks: *Cued recall* – time of test, *F* < 1, *BF_10_* = 0.31; time of test x session, *F* < 1, *BF_10_* = 0.29. *2AFC* – time of test, *F*(1, 32) = 1.80, *p* = .19, η_p_^2^ = .05, *BF_10_* = 0.70; time of test x session, *F* < 1, *BF_10_* = 0.65.

***Summary***

These findings suggest that the time of day at which participants completed the test sessions did not have a significant impact upon their performance in either the training tasks or on their recall and recognition of the nonwords. There was some evidence that only participants who completed the first session in the afternoon showed a significant decrease in RTs in the pause detection task in the second session. However, this was based on overall RTs – there was no interaction with word type (test/control), and no three-way interaction between time of test, session, and word type, suggesting that any differences in RT affected all word types equally.

**Test Location**

Due to parking restrictions at the University of Warwick during term-time, approximately half of the participants were tested in a quiet room in their home (*N* = 20), while the remaining participants were tested in a laboratory at the university (*N* = 16). All participants were tested using the same laptop computer. Test location was not expected to have an effect on the tasks.

***Training Tasks***

Mean accuracy in the phoneme monitoring task was 94.0% (*SD =* 3.2%) in the home group, and 95.0% (*SD* = 2.8%) in the laboratory group. This difference was not significant, *t*(34) = -0.99, *p* = .33, *BF_10_* = 0.46. Mean accuracy in the phoneme segmentation task was 91.4% (*SD =* 7.3%) in the home group, and 90.0% (*SD =* 10.2%) in the laboratory group. This difference was not significant, *t*(34) = 0.49, *p* = .63, *BF_10_* = 0.35.

***Test Tasks***

To examine whether test location impacted upon performance in the test tasks, we re-ran the analyses reported in the main paper, but included test location (home/laboratory) as an additional between-participants variable in each analysis.

For the *pause detection* task, RTs were significantly shorter overall for participants who completed the study in the laboratory, *F*(1, 32) = 7.08, *p* = .01, η_p_^2^ = .18, *BF_10_* = 285.78 (home, *M* = 1222, *SD* = 117; laboratory, *M* = 1107, *SD* = 140). There was also a significant interaction between test location and session, *F*(1,32) = 17.27, *p* < .001, η_p_^2^ = .35, *BF_10_* = 219.32. Additional analyses showed that RTs differed depending on test location in Session 1, *t*(34) = 3.42, *p* = .002, *BF_10_* = 20.83 (home, *M* = 1261, *SD* = 136; laboratory, *M* = 1099, *SD* = 146), but not in Session 2, *t*(34) = 1.64, *p* = .11, *BF_10_* = 0.90 (home, *M* = 1183, *SD* = 105; laboratory, *M* = 1115, *SD* = 142). However, test location did not interact with word type, *F* < 1, *BF_10_* = 0.24, and the three-way interaction between location, session, and word type was not significant, *F*(1, 32) = 1.32, *p* = .26, η_p_^2^ = .04, *BF_10_* = 0.14.

For the *cued recall* and *2AFC* tasks, there were no main effects of test location, and no interactions involving this variable: *Cued recall* – location, *F* < 1, *BF_10_* = 0.34; location x session, *F* < 1, *BF_10_* = 0.40. *2AFC* – location, *F* < 1, *BF_10_* = 0.29; location x session, *F* < 1, *BF_10_* = 0.28.

***Summary***

Test location had no effect on performance in the training tasks or on recall and recognition of the nonwords. There was, however, some evidence that location may have affected the overall speed of responding in the pause detection task. However, this difference had disappeared by Session 2 (which is when we predicted differences between test and control words to emerge, following a period of consolidation), and importantly test location did not interact with word type.

**References**

Brown, H., Weighall, A., Henderson, L. M., & Gaskell, M. G. (2012). Enhanced recognition and recall of new words in 7- and 12-year-olds following a period of offline consolidation. *Journal of Experimental Child Psychology, 122,* 56-72. <http://dx.doi.org/10.1016/j.jecp.2011.11.010>

Gais, S., Lucas, B., & Born, J. (2006). Sleep after learning aids memory recall. *Learning & Memory, 13,* 259-262. <http://dx.doi:10.1101/lm.132106>
